# Intraocular foreign body in the anterior chamber angle of the eye—a 30-year-old ‘emergency’

**DOI:** 10.1093/omcr/omab032

**Published:** 2021-06-18

**Authors:** Nadir A M Ali, Charlotte P Buscombe, David H Jones

**Affiliations:** Ophthalmology Department, Royal Cornwall Hospital, Truro, Cornwall, UK

**Keywords:** penetrating eye injury, intraocular foreign body, hammering, anterior chamber angle

## Abstract

We report an unusual case of a missed intraocular foreign body, which was incidentally discovered in the anterior chamber drainage angle of the left eye of a retired masonry worker, some 30 years after the inciting injury. The ocular penetration and intraocular foreign body were missed during initial emergency management, despite the high-velocity mechanism of chiselling granite, which was reported. This case effectively highlights the need for a careful history and examination in high-velocity injuries to the eye (such as those caused by hammering and grinding), a high index of suspicion for intraocular foreign bodies, and considers best practice in managing such presentations.

## INTRODUCTION

Ocular trauma is an important cause of visual morbidity. An epidemiological study on eye injuries in Scotland reported that, out of 123 penetrating eye injuries included, 30 patients (24.3%) had retained intraocular foreign bodies (IOFBs) [1]. The presence of an intraocular foreign body increases the risk of endophthalmitis and other ocular morbidities like corneal scarring, cataract and retinal detachment [2]. The three most common causes of eye injuries leading to IOFB’s reported are grass trimming, chiselling and hammering, with many injuries potentially avoidable with education surrounding appropriate protective safety glasses [3]. Anterior chamber location of intraocular foreign bodies is rare and usually easily detected on slit-lamp examination [4]. In this report, we present an unusual case of a large anterior segment non-metallic intraocular foreign body that was missed on initial assessment and remained asymptomatic for 30 years, with no effect on visual function.

## CASE REPORT

A 66-year-old retired man was referred as an emergency by his optician for assessment of mild pupil irregularity seen on slit-lamp examination during his routine annual optician review. His past medical history was unremarkable, with no regular medications or known allergies. He is a driver and lives independently with his wife. In the past, he worked as a stonemason and recalled an injury to the left eye while cutting granite using a hammer and a diamond chisel some 30 years ago. At that time, he was assessed in the emergency clinic, reassured that there was no serious ocular injury and discharged from further hospital follow-up with topical antibiotics. He has remained asymptomatic ever since.

**Figure 1:**
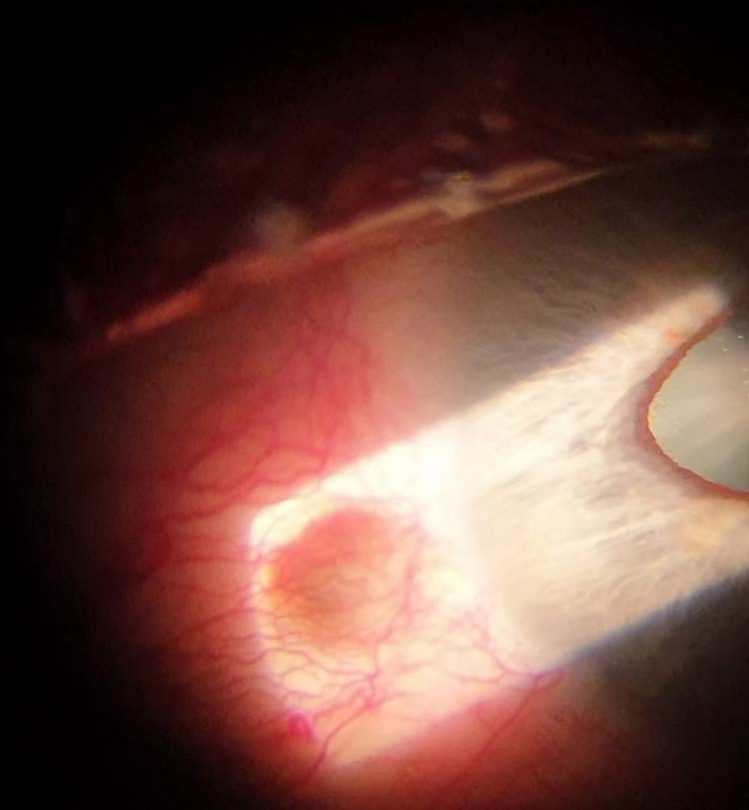
Slit-lamp photo of the site of the intraocular foreign body entry wound and the peaked pupil in the left eye. The foreign body is not visible without the use of gonioscopy.

**Figure 2:**
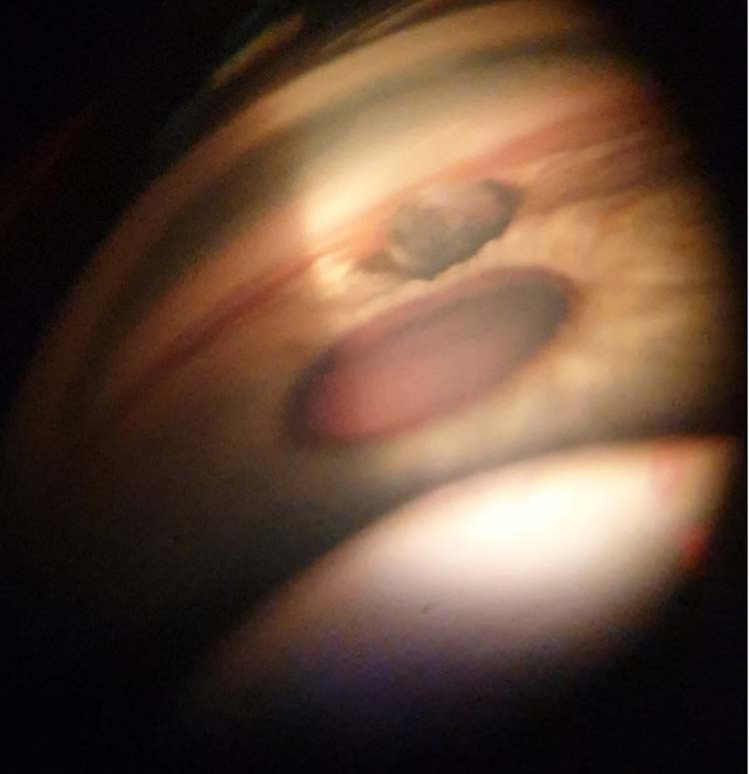
Gonioscopic photo of the foreign body in the nasal angle of the lefteye.

At the time of his recent referral, best-corrected visual acuity was recorded as 6/6 in both eyes with no relative afferent pupillary defect and normal colour vision on Ishihara test. The left eye was white and free of inflammation. However, there was a 2.0-mm, round, brown, subconjunctival, cystic lesion located approximately 1.0 mm posterior to the nasal limbus with corresponding mild peaking of the nasal aspect of the pupil (Fig. 1). The angle of the anterior chamber was examined using a 3-mirror gonio-lens (Volk Optical®, OH, USA). This revealed a large foreign body embedded in the iris close to the nasal angle (Fig. 2). The rest of the left eye examination was normal with clear ocular media and no evidence of optic nerve or retinal dysfunction. The right eye examination was entirely normal.

**Figure 3:**
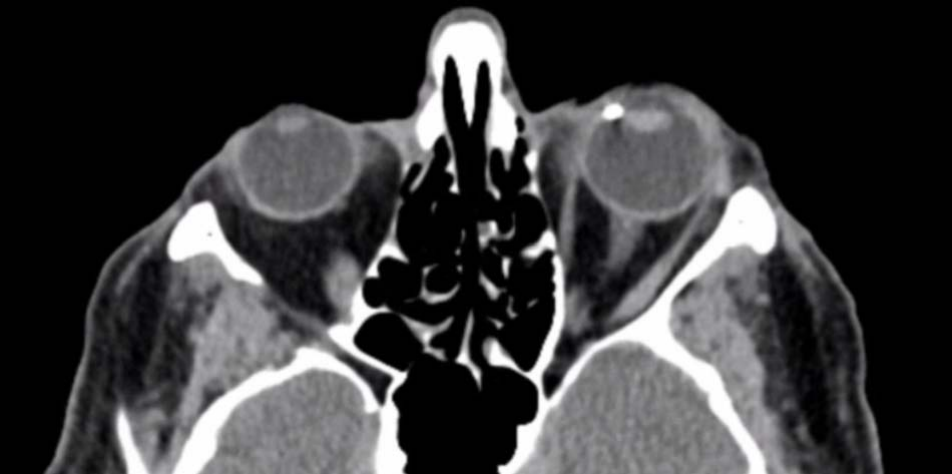
Axial CT scan of the eyes showing a 4-mm dense foreign body in the angle of the anterior chamber of the left eye, located nasally.

A computed tomography (CT) scan confirmed the presence of a 4-mm radio-opaque foreign body in the left eye, located between the lens, the ciliary body and the iris (Fig. 3).

The findings were explained to the patient. As the eye was quiet, the optic nerve and retinal functions were normal, and the foreign body was not causing any symptoms or signs, it was decided to avoid any intervention. The patient was advised to keep annual follow-up with his optician, with re-referral advised in the unlikely event of any subsequent symptoms.

## DISCUSSION

Although thankfully rare in the UK, penetrating ocular trauma and intraocular foreign body constitutes an emergency, which often leads to a poor final best-corrected visual acuity in most patients [3]. The majority of responsible injuries are work related, presenting in young male adults, especially those employed in the construction industry [4, 5].

When evaluating patients with a history of ocular trauma, it is imperative to carefully elicit the mechanism of injury. If a high-velocity mechanism is described, such as hammering, grass mowing, chiselling and grinding, especially without adequate protective goggles, the clinician should have a high index of suspicion for a retained intraocular foreign body and this should always be actively ruled out during the examination and investigations.

Scleral penetrating wounds are occasionally difficult to detect on the slit-lamp. Owing to the nature and the structure of the scleral collagen fibres, high-velocity IOFB’s may enter the eye through smaller wounds in the sclera than their actual diameter. Moreover, they are usually masked by conjunctival chemosis and subconjunctival haemorrhage at the time of injury. Therefore, the presence of conjunctival haemorrhage obscuring the sclera is an examination feature, which should raise clinician suspicion of a scleral entry wound following high-velocity trauma injuries [6].

Orbital radiographs are the most common imaging modality for assessment of suspected IOFBs. Magnetic resonance imaging (MRI) scan should be avoided to reduce the risk of inadvertent damage to the intraocular structures by the movement of a metallic IOFB in response to the strong magnets [7]. Considering the history and the mechanism of the injury, we believe the IOFB was a piece of granite, which is a hard, durable and inert material. This explains the lack of inflammatory reaction to the IOFB. The physical constitution of granite is denser than bone, as evidenced on the CT imaging obtained.

The general rule when managing IOFBs is urgent surgical removal to reduce the risk of blinding complications, such as endophthalmitis and siderosis [8, 9]. In this case, the IOFB was embedded in the anterior chamber’s angle for many years with no functional damage to the surrounding structures and no impact on vision. Due to this innocuous history and the inert nature of the IOFB in our patient, it was felt that any attempt at surgical removal at this late stage may cause more harm than good and the multi-disciplinary decision was therefore to avoid any intervention.

This report highlights some important principles when evaluating ocular trauma. Firstly, the need to take a careful history is key, specifically elucidating the velocity of injury, in order to gauge the likelihood of ocular penetration. When a high-velocity mechanism is elicited the need for appropriate investigation is paramount—this includes a comprehensive detailed ocular examination. If subconjunctival haemorrhage precludes full examination of the sclera, it is prudent to assume an IOFB and obtain radiological imaging, given the significant consequences and increased risks of a missed foreign body. This report also adds to the body of literature, which highlights the need to impress education surrounding occupational hazards and the need to astutely observe health and safety regulations surrounding personal protective equipment, especially in the construction industries.

## CONFLICT OF INTEREST STATEMENT

The authors declare no conflict of interest.

## FUNDING

No sources of funding involved in the preparation or publication process of this manuscript.

## ETHICAL APPROVAL

Ethical approval was not required for this case report.

## CONSENT

A written consent was obtained from the patient to publish the clinical photos and the case report.

## GUARANTORS

Nadir Ali, Charlotte Buscombe and David Jones.
